# Evaluation of the Allplex™ H. pylori & ClariR assay for the detection of clarithromycin-resistant Helicobacter pylori in gastric biopsies

**DOI:** 10.1099/jmm.0.002195

**Published:** 2026-08-24

**Authors:** Callum J. Portman Ross, Katherine Lock, Amaal Ibrahim, Dawn Hedges, Shukri Mohamed, Molly Hales, Takudzwa Edwards, Claire Jenkins, Israel Olonade, Craig Swift

**Affiliations:** 1 UK Health Security Agency, Colindale Avenue, London NW9 5EQ, UK

**Keywords:** clarithromycin, diagnostics, *Helicobacter pylori*, molecular, PCR

## Abstract

**Introduction.** *Helicobacter pylori* colonize the gastrointestinal tract of large proportions of the global population and is associated with the development of gastric ulcers and cancer. Antimicrobial resistance (AMR) to clarithromycin is recognized as a major contributing factor in treatment failure.

**Hypothesis/Gap Statement.** Real-time PCR may be used to detect 23S rRNA gene mutations associated with AMR to clarithromycin in *H. pylori* and has advantages over classical culture and phenotypic susceptibility testing, including no strict requirement for the transport of gastric biopsies.

**Aim.** In this study, we compare the use of the Allplex™ *H. pylori* & ClariR assay (Seegene Inc) for detection of clarithromycin-resistant *H. pylori* with current culture and phenotypic susceptibility testing in 1149 gastric biopsy specimens within our laboratory at the Gastrointestinal Bacteria Reference Unit, UK Health Security Agency.

**Methodology.** PCR was performed using the Allplex™ *H. pylori* & ClariR assay (Seegene Inc) on genomic DNA recovered from gastric biopsy specimens using the MagNA Pure 96 DNA and Viral NA Small Volume Kit and the Pathogen Universal 200 protocol on an MP96 robotic workstation (Roche). Phenotypic susceptibility to clarithromycin was determined by Etest (bioMérieux).

**Results.** We found 94.6% concordance for the presence of either clarithromycin-resistant or -susceptible strains of *H. pylori*.

**Conclusion.** We recommend this assay be included in the UK Health Security Agency (UKHSA) *H. pylori* testing algorithm to improve patient management.

## Introduction

*Helicobacter pylori* are bacteria that commonly reside in the mucus lining of the human stomach. Although most people colonized with *H. pylori* do not experience any symptoms, a small proportion will suffer from indigestion, bloating and/or nausea and may develop gastric or duodenal ulcers. If the ulcers are left untreated, they may bleed or perforate, and more severe symptoms may develop. Furthermore, *H. pylori* is a biological carcinogen and a primary risk factor for gastric cancer and gastric mucosa-associated lymphoid tissue lymphoma [1, 2]. Due to the risks of longer-term sequelae, the diagnosis and treatment of *H. pylori* are recommended in symptomatic patients and in some asymptomatic patients based on risk assessment of familial history of infection (e.g. adenocarcinoma).

Despite declining global rates of infection, studies show that over 40% of the adult population carry *H. pylori* [3], and this has implications for the future burden on healthcare systems from rising levels of antimicrobial resistance (AMR) to secondary incidence of gastric cancer. The fastidious nature of *H. pylori* requires rapid transportation to the laboratory in specialized conditions [4] with samples arriving 24 h after collection being associated with loss of yield. Also, complex culture requirements may limit widespread testing at the local hospital level [5].

Implementation of effective strategies to reduce AMR is a public health priority [6], and the effective stewardship of antimicrobials is paramount. Treatment of *H. pylori* infections involves a minimum of two antimicrobial agents in combination with a proton pump inhibitor. As few agents are effective in the gastric niche, only four antimicrobials, namely, metronidazole, clarithromycin, tetracycline and amoxicillin, are recommended in the UK guidelines for eradication regimens [7]. AMR, particularly to clarithromycin and metronidazole, is a major contributing factor in treatment failure [8].

AMR surveillance of *H. pylori* in the UK is limited, with little or no primary AMR surveillance being conducted at the local level. Gastric biopsies are referred to the UK Health Security Agency (UKHSA) Gastrointestinal Bacterial Reference Unit (GBRU) for detection and antimicrobial susceptibility testing (AST) of *H. pylori* to inform treatment and patient care, with the majority of specimens from patients experiencing first-line treatment failures. Increasing numbers of gastric biopsies referred to GBRU necessitated a reconfiguration of the testing algorithm and the application of molecular methods [9, 10].

This study aimed to assess the performance of the Allplex™ *H. pylori* & ClariR assay (Seegene Inc) and its potential to improve turnaround times and streamline the *H. pylori* diagnostic workflow in a reference laboratory setting.

## Methods

### Specimen submission

As described in the UKHSA Bacteriology Reference Department User Manual (version 17), laboratories referring specimens to the GBRU for testing are advised to perform multiple gastric biopsies (at least two from the antrum and two from the anterior and posterior corpus respectively); to have a treatment-free interval (at least 2 weeks off Proton Pump Inhibiter (PPI) and 4 weeks off antibiotics) before collection; and to send specimens for testing without delay (preferably within 24 h of collection) in a suitable *H. pylori* transport medium or sterile saline.

### Culture and isolation of *H. pylori* from gastric biopsy specimens

Gastric biopsies were plated onto selective *H. pylori* medium (E&O Laboratories Ltd) and non-selective (10% Colombia blood) agar and incubated at 35–38 °C under microaerophilic conditions (4% O_2_, 5% CO_2_, 86% N_2_ and 5% H_2_) in an M45 Microaerophilic Workstation (Don Whitley Scientific, Shipley, UK) for up to 10 days.

### Phenotypic clarithromycin susceptibility testing

Cultures of *H. pylori* were tested alongside the control strain NCTC 12455 for phenotypic susceptibility to clarithromycin using gradient strips E-tests (bioMérieux), which is a testing method that is utilized for determining *H. pylori* susceptibility in other European Reference Laboratories [11]. Inocula were adjusted to McFarland 3 and spread onto the surface of Mueller–Hinton agar with inclusion of 10% blood before gradient strips were applied. Plates were immediately incubated for between 4 and 5 days under microaerophilic conditions (4% O_2_, 5% CO_2_, 86% N_2_ and 5% H_2_) in a M45 Microaerophilic Workstation (Don Whitley Scientific, Shipley, UK). The MICs were interpreted by two independent operators using the 2025 European Committee on Antimicrobial Susceptibility Testing *H. pylori* clinical breakpoints.

### Allplex™ *H. pylori* & ClariR assay (Seegene Inc)

*H. pylori* genomic DNA was released from gastric biopsy material using off-board lysis in 180 µl MagNA Pure DNA Tissue Lysis Buffer (Roche) and 20 µl Proteinase K (New England Biolabs), incubated at 37 °C for 16–24 h. This was followed by DNA recovery and purification using MagNA Pure 96 DNA and Viral NA Small Volume Kit and using the Pathogen Universal 200 protocol on an MP96 robotic workstation with a final elution volume of 100 µl. Eluted DNA was used directly in the Allplex™ *H. pylori* & ClariR assay, and results were interpreted following the manufacturer’s instructions.

## Results

Between 23 June and 22 October 2025, GBRU received 1,149 gastric biopsy specimens for testing for the presence of AMR *H. pylori*, whereas 18/1,149 (1.6%) specimens were received without a given collection date. Of the remaining 1,131 specimens, 686/1131 (60.7%) were received in ≤3 days following collection, whereas 246/1,131 (21.8%) were received in ≤1 day following collection. A total of 445/1,131 (39.3%) specimens were received ≥4 days after collection (Table 1).

**Table 1.** Impact on culture vs PCR results with increasing time from sample collection to date of receipt into GBRU for testing

Days from collection to receipt	PCR +ve		PCR −ve	
	Culture +ve	Culture −ve	Culture +ve	Culture −ve
1 day	152	34	0	60
2 days	152	29	0	94
3 days	103	28	0	34
4 days	55	46	0	78
5 days	31	39	0	24
6 days	29	27	0	14
≥7 days	31	46	0	25
Collection date not specified	4	5	1	8

### Culture vs Allplex™ *H. pylori* & ClariR assay (PCR) for detection of *H. pylori* in gastric biopsy specimens

Overall, 811/1,149 (70.6%) were positive for the presence of *H. pylori* by PCR, and *H. pylori* was cultured from 558/1,149 (48.6%) gastric biopsies tested. *H. pylori* was isolated from one specimen that tested negative for the presence of *H. pylori* by PCR (Table 1). The culture recovered from this biopsy specimen subsequently tested positive for *H. pylori* by PCR, suggesting that low bacterial load may have caused the false-negative results rather than a mutation in the target gene.

**Fig. 1.**
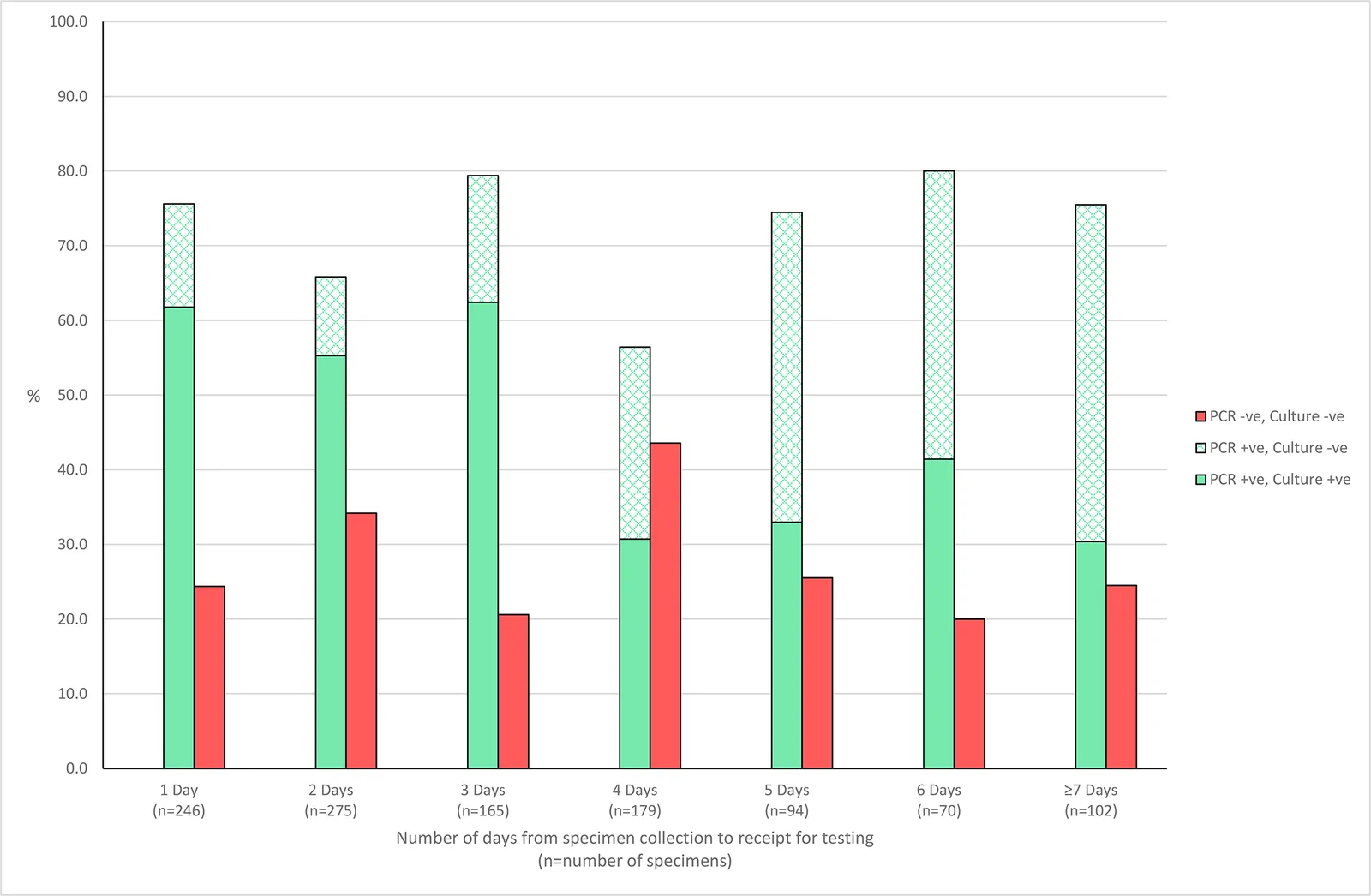
Impact on culture vs PCR results with increasing time from sample collection to date of receipt into GBRU for testing.

An attrition of *H. pylori* recovery by culture from gastric biopsy specimens was seen above 3 days from specimen receipt (Fig. 1).

Of the 686 specimens received ≤3 days from collection, 91 (13.3%) were PCR positive but culture negative, whereas of the 445 specimens received ≥4 days from collection, 158 (35.5%) were PCR positive but culture negative. *H. pylori* was successfully isolated from 407/686 (59.3%) specimens received ≤3 days, in comparison to 146/445 (32.8%) of samples where receipt was ≥4 days. No specimens with a given collection date were found to be PCR negative but culture positive.

### Detection of clarithromycin-resistant *H. pylori* by PCR vs culture

Of the 558 cultures of *H. pylori* that were recovered from gastric biopsies, 555 were included in the evaluation of the PCR detection of clarithromycin resistance compared to phenotypic AST to clarithromycin by E-tests. Two cultures were non-viable on sub-culture, and the third culture was isolated from the specimen that was negative for the presence of *H. pylori* by PCR.

Of the remaining 555 cultures, 525 (94.6%) gave concordant results by PCR from the primary gastric biopsy specimens and AST of the isolates (Table 2). Of these, 402/525 (76.6%) were resistant to clarithromycin by PCR and AST, and 123/525 (23.4%) were susceptible. There were 30/555 (5.4%) discrepant results detected (Table 2), 15/555 (2.7%) of these classified as major errors (i.e. PCR detected a clarithromycin resistant strain of *H. pylori* but only a susceptible strain was recovered from the same specimen) and 15/555 (2.7%) classified as very major errors (i.e. PCR detected a clarithromycin-susceptible strain of *H. pylori* but a resistant strain was recovered from the same specimen). Sensitivity and specificity were calculated to be 96.4 and 81.9%, respectively.

**Table 2.** Comparison of phenotypic (E-test) and genotypic (Allplex™ *H. pylori* & ClariR assay) resistance to clarithromycin

		Allplex™ *H. pylori* & ClariR assay					
		Clarithromycin (23S) mutation detected (resistant)					Clarithromycin (23S) mutation not detected (susceptible)
		A2143G	A2142G	A2142C	A2143G and A2142G	A2143G and A2142C	
Phenotypic susceptibility (E-test)	Resistant	310	70	4	12	6	15
	Susceptible	11	3	1	0	0	123

The most common 23S point mutation identified in 339/417 (81.3%) instances of clarithromycin-resistant detections by PCR was A2143G, whereas the mutation A2142C was only detected in 11/417 (2.6%).

## Discussion

Isolation of *H. pylori* from gastric biopsies is considered the gold standard for diagnosis, essential to investigate antibiotic susceptibility testing and for studying virulence factors to inform vaccine development. However, there are challenges to this approach, including (i) a requirement for rapid transfer from specimen collection to laboratory processing, (ii) special conditions for transporting specimens, (iii) bespoke media and atmospheric growth requirements and (iv) long incubation periods. Most microbiological laboratories in the UK lack the expertise and/or are not equipped to effectively isolate such fastidious bacteria. PCR is an attractive alternative method for detection of AMR *H. pylori*, because it offers (i) increased sensitivity, (ii) is less dependent on rapid transfer of viable specimens to the laboratory and (iii) does not require specialist training, equipment or reagents.

In this study, we performed culture and PCR in parallel to evaluate the advantages and disadvantages of both methods. PCR was more sensitive than culture, and this enhanced sensitivity was more pronounced in those specimens received ≥4 days following collection, with over half of the specimens in this group testing PCR positive but culture negative. This highlights the advantage of PCR over classical culture-based methods for detection of *H. pylori* in gastric biopsy specimens, especially for those samples received ≥4 days. Our study provided further evidence that rapid transportation to the laboratory and timely testing of gastric biopsies is essential for patients failing first line treatment regimens, as for this cohort culture and subsequent phenotypic AST for the wider range of antimicrobials required to inform eradication strategies.

We found very good concordance (94.6%) between PCR detection of clarithromycin resistance from the primary gastric biopsy specimens and AST of isolates from the same specimen. We speculated that the discrepant results were most likely caused by mixed populations of clarithromycin-sensitive and -resistant variants of *H. pylori* within the host. Patients may be colonized for some time prior to the manifestation of symptoms, leading to diverse sub-populations being detected in the gastric mucosa. The scenario where clarithromycin resistance was detected by PCR in the gastric biopsy, but a phenotypically susceptible strain was recovered from the same specimen, may occur where the resistant variant was present in a non-viable state and/or was outcompeted by the sensitive strain during culture.

Where PCR detected a clarithromycin-susceptible strain of *H. pylori*, but a resistant strain was recovered from the same specimen, this may be explained by the resistant strain being present in the specimen below the limit of detection or resistance being attributed to a novel mechanism not covered by the molecular assay.

**Fig. 2.**
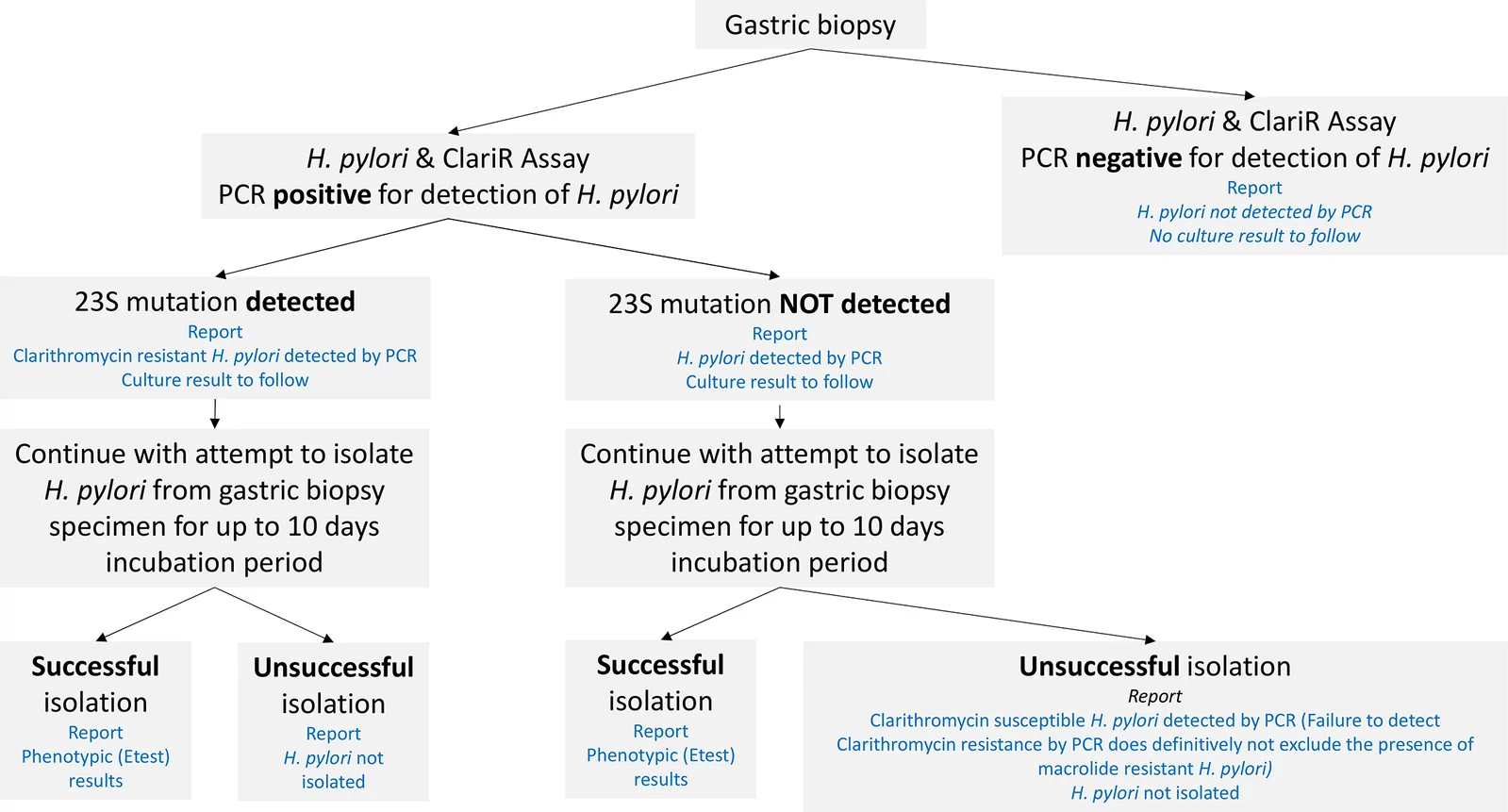
Allplex™ *H. pylori* & ClariR clinical reporting algorithm in GBRU.

In line with previous studies [10, 12–14], when used in a clinical setting, basing treatment regimens on the detection of a clarithromycin-resistant *H. pylori* by the Allplex™ *H. pylori* & ClariR assay is recommended. Even if subsequent culture and AST later identify a clarithromycin-susceptible strain, it is possible that the patient is harbouring a mixed population of susceptible and resistant variants. However, it should be noted that detection of *H. pylori* without clarithromycin resistance by the Allplex™ *H. pylori* & ClariR assay does not definitively exclude the presence of macrolide-resistant *H. pylori*. The clinical reporting algorithm implemented at GBRU following completion of the study described in this report is illustrated in Fig. 2.

Several limitations within the pre-analytical stages of this study were outside the scope of our control including variations in the site and number of gastric biopsies collected from each patient and an inability to govern the environmental and transport factors encountered during ambient temperature transportation of specimens to our laboratory for testing.

## Conclusion

Streamlining pathogen detection using molecular assays enables a more intelligent management of clinical demands, resource allocation and improves result turnaround. Inclusion of the Allplex™ *H. pylori* & ClariR assay into *H. pylori* testing algorithms reliant on culture alone may better support patient management, particularly in settings where there are significant delays between specimen collection and culture. However, PCR may prove limited in its use for treatment refractory patients failing triple therapy with inclusion of clarithromycin. In these cases, susceptibility testing for other antimicrobial agents, particularly levofloxacin and tetracycline, may still be required to achieve successful eradication.

## Abbreviations

AMR: antimicrobial resistance
AST: antimicrobial susceptibility testing
GBRU: Gastrointestinal Bacterial Reference Unit
PPI: proton pump inhibiter
UKHSA: UK Health Security Agency
